# Maternal occupational noise exposure during pregnancy and children’s early language acquisition

**DOI:** 10.1371/journal.pone.0301144

**Published:** 2024-04-16

**Authors:** Soile Jungewelter, Helena Taskinen, Markku Sallmén, Marja-Liisa Lindbohm, Erkko Airo, Jouko Remes, Minna Huotilainen, Eira Jansson-Verkasalo

**Affiliations:** 1 Finnish Institute of Occupational Health, Helsinki, Finland; 2 University of Helsinki, Helsinki, Finland; 3 University of Tampere, Tampere, Finland; 4 Institute for Behavioral Sciences, University of Helsinki, Cognitive Brain Research Unit, Helsinki, Finland; 5 Department of Psychology and Speech-Language Pathology, University of Turku, Turku, Finland; Tulane University School of Public Health and Tropical Medicine, UNITED STATES

## Abstract

**Introduction:**

Noise exposure during pregnancy may affect a child’s auditory system, which may disturb fetal learning and language development. We examined the impact of occupational noise exposure during pregnancy on children’s language acquisition at the age of one.

**Methods:**

A cohort study was conducted among women working in the food industry, as kindergarten teachers, musicians, dental nurses, or pharmacists who had a child aged <1 year. The analyses covered 408 mother-child pairs. Language acquisition was measured using the Infant-Toddler Checklist. An occupational hygienist assessed noise exposure individually as no (N = 180), low (70–78 dB; N = 108) or moderate/high exposure (>79 dB; N = 120).

**Results:**

Among the boys, the adjusted mean differences in language acquisition scores were -0.4 (95% CI -2.5, 1.8) for low, and -0.7 (95% CI -2.9, 1.4) for moderate/high exposure compared to no exposure. Among the girls the respective scores were +0.1 (95% CI -2.2, 2.5) and -0.1 (95% CI -2.3, 2.2). Among the children of kindergarten teachers, who were mainly exposed to human noise, low or moderate exposure was associated with lower language acquisition scores. The adjusted mean differences were -3.8 (95% CI -7.2, -0.4) for low and -4.9 (95% CI -8.6, -1.2) for moderate exposure.

**Conclusions:**

In general, we did not detect an association between maternal noise exposure and children’s language acquisition among one-year-old children. However, the children of kindergarten teachers exposed to human noise had lower language acquisition scores than the children of the non-exposed participants. These suggestive findings merit further investigation by level and type of exposure.

## Introduction

Noise is known to penetrate the tissues and fluids surrounding the fetal head and to stimulate the inner ear through bone conduction. The abdominal wall and uterus attenuate some noises, allowing the fetus to hear predominately low-frequency sounds. High-frequency sounds in turn are greatly attenuated by the abdomen [1,2].

Environmental factors play an important role in the development of a child’s auditory processing and language acquisition. Auditory processing develops early, and fetuses are able to discriminate between the sounds that they hear [3].

Studies of rats have shown that compared to music, prenatal noise weakens brain development and therefore possibly also the development of the auditory system [4,5].

Findings among humans have indicated delays in the language development of prematurely born infants who have spent their first weeks (last trimester) in the sound environment of neonatal intensive care units. Some researchers have proposed that these delays could partially be related to differences in the sound environment, as these units typically offer more machine-like sounds than language-related sounds [6–8].

A study of neonates has shown that acoustic noise changes the processing of sounds in the brain [9]. Another study has revealed that compared to silence, short-term exposure of two-year-old children to acoustic noise affects the brain processes related to sound processing [10]. This may in turn affect a child’s language acquisition, and possibly lead to a weakened ability to learn concepts [11], which is detrimental to the overall ability to learn. Such damage might limit a child’s ability to study, graduate and build a successful career as an adult.

Several environmental factors have been observed as affecting a child’s language acquisition. During the first year, children usually learn to comprehend words and speech, and to use the sounds they hear in their babbling and their first words [12]. Listening to music and a mother’s story-telling supports language acquisition, whereas background noise and the insensitiveness of a mother have been linked to delayed language acquisition [13–17]. Neurally, noise degrades central auditory processing [18], which is fundamental for language acquisition [11,12]. Delayed early language acquisition is a substantial risk factor for later language impairment [19].

The findings regarding the effect of prenatal noise exposure on hearing impairments among children have been conflicting. Two early reports [20,21] associated maternal exposure to high noise levels (85–95 dB or >100 dB) with hearing deterioration among children, but the studies were based on small samples and therefore had low precision. A later study [22] indicated no hearing impairment after exposure (>80 but <90 dB) but presented unspecific inclusion criteria. A recently published study [23], however, showed an association between occupational noise exposure (>85 dB) during pregnancy and hearing dysfunction among children.

We hypothesized that regular exposure to noise during pregnancy might affect the fetal auditory system in ways that could influence language development. Thus, the aim of our study was to investigate the potential impact of a mother’s occupational noise exposure during pregnancy on the language acquisition of her child. We investigated whether prenatal exposures to industrial noise, high frequency noise in dental clinics, human noise in kindergartens, and musical sounds in musicians’ work affect children’s language acquisition at the age of one. The impact of maternal noise exposure during pregnancy on a child’s language acquisition has not been studied previously.

## Material and methods

### Participants and data collection procedure

**Fig 1:**
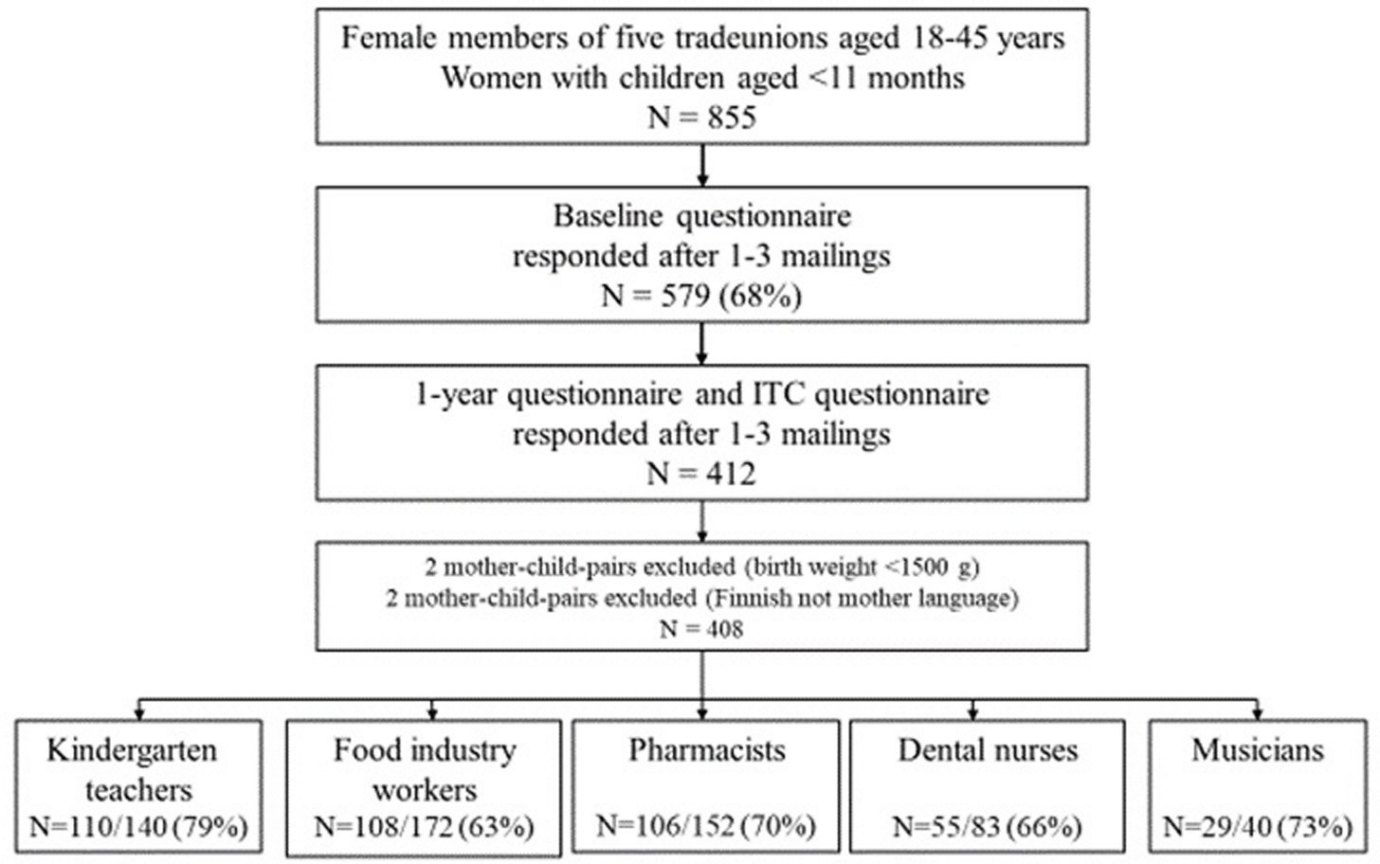
Flow chart of data collection and participation at baseline and when child was one (ITC = Infant-Toddler Checklist). Percentages in brackets (%) indicate proportion of women who returned questionnaire.

The study population consisted of Finnish mothers working in sectors with or without occupational noise exposure and their children born between April 2013 and March 2014. We focused on occupational branches in which exposure to various types of noise was known to be present, and in which documented information on the range of noise or sound level in Finnish workplaces was available. Pharmacists were selected as the non-exposed control group. In Finland, 70% of working aged women were members of a trade union in 2013 (Ministry of Employment and the Economy). We identified 17 138 women at a fertile age of 18–45 from the members of five trade unions: kindergarten teachers (N = 3900), food industry workers (N = 6395), musicians (N = 937), dental nurses (N = 3283), and pharmacists (N = 2623). Using the personal identity codes of the women, we collected the data (birth date, gender, native language) on their children (singletons) between the ages of 6 weeks and 11 months from the Population Register Centre. On the mother, we collected the following information: native language, address, and ages of all biological children. We restricted the population to Finnish-speaking mothers, and obtained data on 187 kindergarten teachers, 291 food industry workers, 48 musicians, 137 dental nurses, and 192 pharmacists: 855 mother-child pairs in total. Data on exposure and language acquisition were collected using several questionnaires, in two phases (Fig 1).

#### Phase 1

The Baseline questionnaire was sent at the same time to all the 855 women found from among the five trade union members. The children were then between 6 weeks and 11 months old. The questionnaire focused on background factors such as maternal employment, detailed work task descriptions, and exposure to occupational noise and stress during pregnancy. The questions on noise were a) “During your pregnancy, did the noise in your working environment disturb normal conversation?”, b) “During your pregnany, was the noise in your work environment so loud that you could not hear normal speech from one meter’s distance?”. The weekly duration of such noise and the use of hearing protection devices were also elicted. These kinds of questions have been found to be practical for estimating noise level [24]. A study of construction workers by Neitzel [25] showed that trade was a poor predictor of noise exposure, whereas an internal validation study indicated good agreement between worker self-reporting and researcher observation. We also requested information on exposure to high-level leisure-time noise, its frequency (daily/weekly/more seldom) and the activity that caused it. Furthermore, we asked whether the women experienced stress (on a five-point Likert scale varying from 1 “not at all” to 5 “very much”) during pregnancy and after birth. This question has shown to be valid for group-level analysis [26].

#### Phase 2a

The One-year background questionnaire included questions on the health (chronic diseases diagnosed by a physician before, during and after pregnancy as well as potential hearing problems) of the mother. It had detailed questions on the routine hearing tests (otoacoustic emission measurements) of the newborn child, ear infections, hospital treatments, malformations, and developmental disorders or other diseases or injuries during the child’s first year of life. We also asked about possible day care, and the mothers’ lifestyle factors (smoking or alcohol use). Communication between the mother and the child (frequency and duration of talking, reading and singing to the child, and the group activities of the child) was covered in detail in the questionnaire. Possible familiar first and second degree language acquisition disorders among relatives were elicited. We also asked about parental education (low = 9 years primary school; mid-level = high school or vocational school; high = university or university of applied sciences), and mother’s exposure to ototoxic substances such as solvents, metals, or other chemicals.

#### Phase 2b

Data on the language acquisition of the child at the age of one were collected using the Finnish version of the Infant-Toddler Checklist (ITC) [27] called Esikko [28]. The ITC consists of 24 items (questions) organized into seven cluster areas that form three composites: social communication (expression of motions and eye gaze, communication, gestures), speech production (sounds, words) and language comprehension (language comprehension, object use). The maximum scores of the composites are: 26 for social communication, 14 for speech production, and 17 for language comprehension (57 in total). A higher score indicates a higher functioning. Total scores below 28 are typically referred to additional follow up consultations.

The Baseline questionnaire was sent to the 855 female trade union members three weeks to 11 months before their children turned one. Altogether 579 (68%) responded (Fig 1): 77% of the kindergarten teachers, 59% of the food industry workers, 79% of the dental nurses, 83% of the musicians, and 79% of the pharmacists. We sent the One-year background enquiry and ITC questionnaire (paper or web-based) to those who responded when their children turned one. Two reminders were sent if necessary. Altogether 412 (71% of baseline respondents and 48% of all the 855 women) women responded to both the One-year background questionnaire and the ITC questionnaire when their child was aged one year ± 1 weeks; 79% of the kindergarten teachers, 63% of the food industry workers, 66% of the dental nurses, 73% of the musicians, and 70% of the pharmacists. Children with a birth weight of <1500 g (N = 2) and women with a mother tongue other than Finnish (N = 2) were excluded. The final analyses consisted of 408 mother-child pairs.

### Exposure assessment

An experienced occupational hygienist assessed the noise exposure of the women during pregnancy by combining existing information on noise exposure at Finnish workplaces in their professions and the questionnaire data. The range of exposure and workplace noise levels in the target professional groups are well known and documented. We used both published research data and results from The Finnish Database of Occupational Exposure Measurements (FDOEM) maintained by the Finnish Insitute of Occupational Health [29,30]. The questionnaires, in turn, elicited information on how the respondents were exposed to noise during pregnancy. The duration (no noise, noise <2 hours, 2–6 hours, 6–10 hours, >10 hours per week) of noisy events that disturbed or blocked speech communication (impossible to hear speech from a distance of one meter), type of identified noise sources at work, and the respondents’ use of hearing protection were also used to help place each individual in the most plausible category within each occupational group. Noisy activities during leisure time were queried and classified separately. Exposure assessment was made blindly, without knowledge of the language acquisition of the child.

Exposure assessment among kindergarten teachers was based on two sets of data. In a FIOH research project in 25 randomly selected kindergartens, the range of daily noise exposure levels was 71–84 dB, and only in two of the 50 individual measurements did the level exceed 80 dB [29]. Thus, the majority of the kindergarten teachers was classified into the low-exposure category. However, in kindergartens in which the personnel reported disturbing noise and measurements were conducted (FDOEM), 67% of personal noise exposure measurements exceeded 80 dB. Therefore, teachers reporting weekly exposure to noise events that hindered communication were placed into the moderate exposure class.

Food industry work environments ranged from bakeries to food factories. The questionnaire asked about the individual type of work (bakery, meat processing, food service, packing, box washing line) and produced items. In previous measurements, exposure had ranged from low to >85 dB (FDOEM).

In dental care, noise producing equipment are: high-/slow-speed hand pieces, suction tubes, ultrasonic scalers, and compressed air. The exposure assessment was based on self-reported tool usage, and noise levels were reported elsewhere. In Finland, typical dental tool noise level has been measured as 78–83 dB, but internationally higher noise levels have also been reported [31,32].

We asked the musicians about their main and side instrument as well as singing, the size of their orchestra or group, type of music (classical, rock, chorus), and the weekly time of rehearsal, performances, and possible teaching lessons. Several projects have assessed musicians’ noise exposure in Finnish professional orchestras and musical education. The sound level for symphony orchestra musicians is 83–96 dB, depending on instrument and position [30]. The majority of the musicians in our data were classical orchestra musicians. The others were either full-time or semi-professional performers of popular music genres. The sound level of amplified music during practice and performance was assumed to exceed 90 dB on the basis of measurements during concerts, in which levels of 95–105 dB have been registered (FDOEM).

Based on the questionnaires, the pharmacists were assessed as non-exposed. Occasional repair work nearby, for example, could cause low exposure.

Occupational noise exposure (the highest exposure level in any pregnancy trimester) was classified into one of the four categories:

1. Non-exposed: Environmental sounds come mostly from speaking, normal office equipment or home sounds.
2. Low exposure: The presence of environmental noise becomes noticeable and requires the use of a raised voice during conversation. Noise sources in these environments can be, for example, groups of people talking loudly, or quiet machinery or brief occurences of higher level sounds. The approximate range of noise exposure level in this category is 70–78 dB.
3. Moderate exposure: Noise and loud sounds make conversation difficult but do not block it. The approximate range of daily noise exposure level is 79–84 dB. Many of these workers consider hearing protection useful.
4. High exposure: The sound or noise level is so high that conversation is difficult or blocked most of the working time, and the risk of hearing loss is high. Typical noise exposure is >85 dB, and the use of hearing protection is mandatory.

### Statistical analyses

We compared the language acquisition of children of mothers exposed to noise at work during pregnancy to the language acquistion of children of non-exposed working mothers. As a measure of language acquisition, we used the summary score of the ITC questionnaire and the scores of its three composites: social communication, speech production and language comprehension.

We used multivariable linear regression to assess the mean differences between the language acquisition scores in the occupational exposure categories. Statistical significance was determined by 95% confidence intervals (CI). Missing ITC score data were replaced by the mean values of each occupational group (one dental nurse and one pharmacist) in the single items. The analyses were performed using SAS 9.4^®^ and IBM SPSS Statistics 23^®^.

Due to the small number of women in the highest category, the two highest noise exposure categories, moderate and high, were combined for statistical analyses. The exposed and non-exposed women in the four occupational groups were also analyzed separately, but not the musicians, because of their small number. Women who were not working during pregnancy (N = 54) or did not work in the occupation corresponding to their trade union (9 dental nurses and 3 food industry workers) were excluded from these analyses. One musician and one dental nurse who worked in a kindergarten were transferred to the kindergarten teacher group.

On the basis of earlier studies, we identified a number of potential confounders on factors which may affect a child’s language acquisition. The factors that were associated with language acquisition in our data, and which were included in the final multivariable models as potential confounders were maternal age (20–34 and 35–44 years), talking (<1 hour/day, ≥1 hour/day), looking at and reading books (not yet or occasionally, a few times a week, daily) and singing to the child (never or occasionally, a few times a week, daily). The boys and girls were analyzed separately, as their language acquisition scores clearly differed. However, we adjusted for gender in the analyses by occupational group. Other potential confounding factors, such as parental education, mother’s use of alcohol, diabetes mellitus during pregnancy, history of acute otitis media, number of siblings, or language disorders among close relatives were not associated with the child’s language acquisition and were therefore not included in the final models.

The following health conditions were very rare in our data (<5% of the observations) and were not included in the final models: preterm birth, low birth weight, low APGAR score, congenital abnormalities or postnatal diseases requiring hospital treatment, child’s hearing loss, abnormal results in otoacoustic emission measurements as newborn (3 children), and mother’s chronic diseases (mental disorders, hyperthyreosis or hypothyreosis, diabetes mellitus, or other serious illness diagnosed by a physician), maternal hepatogestosis, and smoking. The same applies to noise exposure during leisure time, because it was not associated with language acquisition. Most children were taken care of at home (92.4%); only 7.6% (N = 31) outside the home. Thus, for the majority, the noise was at normal family-life level.

### Ethics approval

The research plan was approved by the Coordinating Ethical Committee of the Hospital District of Helsinki and Uusimaa (approval number 54/13/03/00/13).

## Results

About 87% (N = 354) of the mothers worked during their pregnancy. About 36% of these mothers were not exposed to occupational noise, 31% were exposed to low levels of noise, 21% to moderate noise, and 12% to high-level noise. Only the musicians and food industry workers were exposed to high-level noise.

The group of children of highly exposed mothers included more girls (66%) than boys (34%), whereas the sex ratio in other groups was even. Table 1 shows further background characteristics by levels of noise exposure.

**Table 1:** Characteristics of study participants by estimated occupational noise exposure during pregnancy.

	Occupational noise exposure			
Characteristics	No exposure	Low exposure	Moderate exposure	High exposure
	N (%)	N (%)	N (%)	N (%)
**Occupational group**				
Kindergarten teachers	15 (8)	52 (48)	28 (37)	0 (0)
Musicians	2 (1)	3 (3)	7 (9)	16 (36)
Food industry workers	14 (8)	19 (8)	25 (33)	28 (64)
Dental nurses	13 (7)	20 (19)	16 (21)	0 (0)
Pharmacists	82 (46)	14 (13)	0 (0)	0 (0)
Unemployed	54 (30)	-	-	-
Total	180	108	76	44
**Child’s mother**				
*Maternal age at birth (years)*				
≤35	147 (82)	93 (86)	64 (84)	37 (84)
>35	33 (18)	15 (14)	12 (16)	7 (16)
*Maternal education*				
Low	1 (1)	0 (0)	3 (4)	3 (7)
Mid-level	45 (25)	26 (24)	31 (41)	23 (52)
High	134 (74)	81 (75)	41 (54)	18 (41)
Unknown	0 (0)	1 (1)	1 (1)	0 (0)
*Smoking during pregnancy*				
Yes	6 (3)	6 (6)	3 (4)	6 (14)
No	174 (97)	101 (94)	72 (95)	38 (86)
Unknown	0 (0)	1 (1)	1 (1)	0 (0)
*Alcohol use during pregnancy*				
Yes	14 (8)	7 (7)	5 (7)	3 (7)
No	166 (92)	101 (94)	71 (93)	41 (93)
*Stress during pregnancy*				
Not at all	22 (12)	17 (16)	10 (13)	4 (9)
Only a little	78 (43)	41 (38)	34 (45)	22 (50)
Some	62 (34)	38 (35)	25 (33)	11 (25)
Rather much/very much	18 (10)	12 (11)	7 (9)	7 (16)
**Child’s father**				
*Paternal education*				
Low	4 (2)	3 (3)	5 (7)	1 (2)
Mid-level	87 (48)	58 (54)	33 (43)	25 (57)
High	86 (48)	45 (42)	36 (47)	17 (39)
Unknown	3 (2)	2 (2)	2 (3)	1 (2)
**Child**				
*Gender*				
Female	87 (48)	54 (50)	38 (50)	29 (66)
Male	93 (52)	54 (50)	38 (50)	15 (34)
*Gestational age (weeks)*				
**≥**37	178 (99)	102 (94)	69 (91)	40 (91)
<37	1 (1)	4 (4)	3 (4)	3 (7)
Unknown	1 (1)	2 (2)	4 (5)	1 (2)
*Birth weight (grams)*				
<2500	1 (1)	2 (2)	2 (3)	1 (2)
<4500 but ≥2500	174 (97)	101 (94)	66 (87)	42 (96)
≥4500	5 (3)	4 (4)	8 (11)	1 (2)
Unknown	0 (0)	1 (1)	0 (0)	0 (0)
*Acute otitis media*				
0–2	164 (91)	104 (96)	70 (92)	41 (93)
≥3	15 (8)	4 (4)	5 (7)	3 (7)
Unknown	1 (1)	0 (0)	1 (1)	0 (0)
*Congenital abnormalities*				
No	175 (93)	105 (97)	72 (95)	44 (100)
Yes	5 (3)	3 (3)	4 (5)	0 (0)
*Language disorders in close relatives*				
No	167 (93)	97 (90)	70 (92)	39 (89)
Yes	10 (6)	10 (9)	5 (7)	5 (11)
Unknown	3 (2)	1 (1)	1 (1)	0 (0)
*Siblings*				
0	66 (37)	45 (42)	39 (51)	25 (57)
1	70 (39)	38 (35)	23 (30)	13 (30)
>2	44 (24)	25 (23)	14 (18)	6 (14)
*Child at day care*				
No	169 (94)	98 (91)	67 (88)	41 (93)
Yes	9 (5)	10 (9)	9 (12)	3 (7)
Unknown	2 (1)	0 (0)	0 (0)	0 (0)

Time spent talking to their children was shorter among the non-exposed mothers than among the other mothers (Table 2). 46% read daily to their children. There were no systematic differences in terms of looking at and reading books between the exposure categories. The moderately or highly exposed mothers sang more often to their children than the mothers in other groups. There were no clear differences in terms of attending musical play school or other activities between the exposure groups.

**Table 2:** Communication between mother and child and child’s activities by estimated occupational noise exposure during pregnancy.

	Occupational noise exposure			
Communication and activities	No exposure	Low exposure	Moderate exposure	High exposure
	N (%)	N (%)	N (%)	N (%)
*Talking to child*				
Daily, <1 hour	39 (22)	13 (12)	5 (7)	6 (14)
Daily, ≥1 hour	140 (78)	94 (87)	71 (93)	38 (86)
Unknown	1 (1)	1 (1)	0 (0)	0 (0)
*Looking at or reading books to child*				
Not yet or occasionally	49 (27)	21 (19)	12 (16)	15 (34)
A few times a week	54 (30)	29 (27)	33 (43)	6 (14)
Daily	77 (43)	55 (51)	31 (41)	23 (52)
Unknown	0 (0)	3 (3)	0 (0)	0 (0)
*Singing to child*				
Never or occasionally	16 (9)	14 (13)	8 (11)	2 (5)
A few times a week	38 (21)	26 (24)	10 (13)	8 (18)
Daily	124 (69)	68 (63)	58 (76)	34 (77)
Unknown	2 (1)	0 (0)	0 (0)	0 (0)
*Child attends musical playschool*				
Yes	49 (27)	25 (23)	20 (26)	12 (27)
No	131 (73)	83 (77)	56 (74)	32 (73)
*Child participates in activities other than musical playschool*				
Yes	56 (31)	30 (28)	19 (25)	14 (32)
No	124 (69)	78 (72)	57 (75)	30 (68)
*Child participates in both musical playschool and other activities*				
Yes	25 (14)	8 (7)	8 (11)	7 (16)
No	155 (86)	100 (93)	68 (90)	37 (84)

There were no statistically significant differences between the language acquisition total scores of the boys or girls according to maternal exposure categories (Table 3). The girls achieved significantly higher language acquisition mean scores (35.8) than the boys (33.3) (*P*<0.001). Among the boys, the adjusted mean differences in ITC scores were -0.4 (95% CI -2.5, 1.8) for low exposure, and -0.7 (95% CI -2.9, 1.4) for moderate/high exposure, in comparison to the non-exposed. Among the girls these were +0.1 (95% CI -2.2, 2.5) and -0.1 (95% CI -2.3, 2.2), respectively (Table 3). Among the boys, reading and speaking to one’s child was associated with high language acquisition scores, as well as maternal age at birth (<35 years) and working status during pregnancy. Among the girls, only reading was related to high scores.

**Table 3:** Crude and adjusted mean differences between ITC total language acquisition scores of one-year-old boys and girls by estimated noise exposure during pregnancy. Adjusted for mother’s age at birth; working status during pregnancy; and talking, reading and singing to one’s child. A multivariable model.

	Boys						Girls					
	N	Mean score	Mean differences between ITC scores				N	Mean score	Mean differences between ITC scores			
Variable			Crude	Adjusted	95% CI	*P* value			Crude	Adjusted	95% CI	*P* value
	
*Occupational noise exposure*												
No exposure	68	32.6				Reference	58	35.6				Reference
Low exposure	54	33.7	+1,1	-0.4	-2.5, 1.8	0.724	54	36.0	+0.4	+0.1	-2.2, 2.5	0.912
Moderate/high exposure	53	34.0	+1.4	-0.7	-2.9, 1.4	0.498	67	35.8	+0.2	-0.1	-2.3, 2.2	0.962
*Mother’s working status during pregnancy*												
No	25	30.6				Reference	29	34.8				Reference
Yes	175	33.7	+3.1	+2.9	0.2, 5.6	0.035	179	35.9	+1.1	+0.7	-2.2, 3.6	0.631
*Maternal age at birth*												
≥35	41	31.0				Reference	47	35.9				Reference
<35	159	33.9	+2.9	+2.9	0.9, 4.9	0.005	161	35.7	-0.2	-0.2	-2.3, 1.9	0.861
*Reading to child*												
Occasionally	53	30.0				Reference	44	32.9				Reference
A few times a week	59	34.0	+4.0	+3.6	1.4, 5.9	0.002	66	34.9	+2.0	+1.5	-1.0, 4.0	0.239
Daily	88	34.8	+4.8	+3.8	1.6, 6.0	0.001	98	37.6	+4.7	+3.6	1.1, 6.0	0.004
*Singing to child*												
Occasionally	17	30.6				Reference	23	32.7				Reference
A few times a week	41	33.3	+2.7	+1.3	-2.1, 4.7	0.460	43	34.0	+1.3	+0.5	-2.8, 3.9	0.745
Daily	142	33.6	+3.0	+1.1	-2.0, 4.3	0.478	142	36.8	+4.1	+2.6	-0.4, 5.6	0.087
*Talking to child*												
Daily, <1 hour	31	29.7				Reference	32	33.5				Reference
Daily, ≥1 hour	169	33.9	+4.2	+3.0	0.6, 5.4	0.015	176	36.2	+2.7	+1.5	-1.0, 4.0	0.231

No clear associations were found between noise exposure and the different composites of language acquisition including child’s social communication, speech production and language comprehension (Table 4).

**Table 4:** Crude and adjusted mean differences between ITC scores in three composites of language acquisition of one-year-old boys and girls by estimated noise exposure during pregnancy. Adjusted for mother’s age at birth; working status during pregnancy; and talking, reading and singing to one’s child.

	Boys					Girls				
Composites of language acquisition by noise exposure	Mean score	Mean differences between ITC scores				Mean score	Mean differences between ITC scores			
		Crude	Adjusted	95% CI	*P* value		Crude	Adjusted	95% CI	*P* value
	
*Social communication*										
No exposure	17.8				Reference	18.9				Reference
Low exposure	18.1	+0.3	-0.5	-1.7, 0.7	0.376	19.2	+0.3	-0.1	-1.4, 1.1	0.814
Moderate/high exposure	18.4	+0.6	-0.4	-1.6, 0.8	0.513	19.2	+0.3	-0.03	-1.2, 1.1	0.961
*Speech production*										
No exposure	5.8				Reference	6.9				Reference
Low exposure	6.4	+0.6	+0.2	-0.6, 1.1	0.615	7.4	+0.5	+0.7	-0.2, 1.6	0.139
Moderate/high exposure	6.6	+0.8	+0.1	-0.7, 1.0	0.778	6.8	-0.1	-0.003	-0.9, 0.9	0.995
*Language comprehension*										
No exposure	9.0				Reference	9.8				Reference
Low exposure	9.2	+0.2	-0.1	-0.8, 0.7	0.868	9.4	-0.4	-0.4	-1.3, 0.5	0.372
Moderate/high exposure	8.9	-0.1	-0.5	-1.2, 0.3	0.205	9.7	-0.1	-0.02	-0.9, 0.8	0.956

Analyses by occupational group were restricted to the mothers who worked during pregnancy (N = 354) (Table 5). Compared to no exposure, low or moderate noise exposure was associated with lower language acquisition scores among the children of kindergarten teachers, and with language comprehension in particular. The adjusted mean score of this composite was 9.2 (mean difference -1.3 (95% CI -2.5, -0.1, *P*<0.05)) for low exposure and 9.1 (mean difference -1.5, 95% CI -2.8, -0.2, *P*<0.05) for moderate exposure. The mean score for the non-exposed was 10.5 in comparison.

**Table 5:** Adjusted mean differences between ITC total scores of one-year-old children of employed mothers by estimated noise exposure and mother’s occupation during pregnancy. ^1^Adjusted for gender of child. ^2^Adjusted for gender of child; mother’s age at birth; and talking, reading and singing to one’s child. ITC total score range 0–57.

		ITC score	Mean differences between ITC scores			ITC score	Mean differences between ITC scores		
Noise exposure by occupational group	N	Adjusted^1^ mean	Adjusted^1^	95% CI	*P* value	Adjusted^2^ mean	Adjusted^2^	95% CI	*P* value
*Kindergarten teachers*									
No exposure	15	39.5	Reference			37.3	Reference		
Low exposure	53	35.8	-3.7	-7.0, -0.4	0.030	33.5	-3.8	-7.2, -0.4	0.030
Moderate exposure	29	34.8	-4.7	-8.3, -1.1	0.012	32.4	-4.9	-8.6, -1.2	0.010
*Food industry workers*									
No exposure	12	32.9	Reference			31.3	Reference		
Low exposure	19	33.9	+1.0	-3.8, +5.7	0.689	32.6	+1.3	-3.6, +6.2	0.593
Moderate/high exposure	52	34.1	+1.2	-2.9, +5.3	0.561	32.8	+1.5	-2.8, +5.8	0.482
*Dental nurses*									
No exposure	8	38.8	Reference			36.7	Reference		
Low exposure	15	33.6	-5.1	-10.2, -0.01	0.050	33.6	-3.1	-8.1, +1.8	0.205
Moderate exposure	16	35.5	-3.2	-8.3, +1.9	0.205	34.0	-2.7	-7.4, +2.1	0.264
*Pharmacists*									
No exposure	82	33.6	Reference			31.3	Reference		
Low exposure	14	33.3	-0.4	-4.0, +3.3	0.847	31.1	-0.2	-3.8, +3.4	0.903

To test whether these associations could be explained by stress, as a sensitivity analysis we included maternal stress during pregnancy in the models. The results were virtually unchanged in terms of the total and composite scores. No associations between noise exposure and language acquisition were seen among the food industry workers. The children of the musicians had a relatively high mean total score (35.6). Due to small numbers, it was not possible to analyze their language acquisition by noise level.

## Discussion

### Principal findings

We found no clear association between maternal occupational noise exposure to low exposure (approximately 70–78 dB) or moderate and high exposure (approximately >79 dB) and the child’s language acquisition at one year of age in the entire data. The results were similar when we studied the three different composites of language acquisition: social communication, speech production and language comprehension. Accordingly, industrial noise among food industry workers or noise in dental clinics showed no association with children’s language acquisition.

No previous studies have examined the effects of prenatal occupational noise exposure on the language acquisition of children at one year of age. Earlier studies have focused on prenatal noise exposure effects on hearing impairments among children [20–23], and the findings of these studies have been conflicting. Some studies have associated maternal exposure to high noise levels (>85 dB) with hearing deterioration among children, whereas others have indicated no hearing impairment after exposure.

Among the children of the kindergarten teachers, who were mainly exposed to human noise, low or moderate noise exposure was associated with lower language acquisition scores. In addition to noise, stress during pregnancy might be a potential risk factor for lower language acquisition. Laplante [33] observed that stress during pregnancy affects the language functioning of human toddlers. Stress could be either a confounding factor or an intermediate variable in the causal pathway from noise exposure to language acquisition. However, our analyses indicated no role for stress because the association of noise with children’s language acquisition was virtually unchanged after adjustment for maternal stress during pregnancy.

Most of the musicians were exposed to moderate or high noise levels, but the ITC scores for their children were relatively high. Earlier studies [34] have shown music to have positive effects on language acquisition. Accordingly, a number of studies have revealed that children who undergo music training have stronger cognitive linguistic abilities, for example in vocabulary and perception, than children with no musical training [16].

Noise exposure in different trimesters may have different effects in the child’s later development, but studies of the effects of noise on the human fetus during different stages of pregnancy are lacking. Some studies of pregnant animals have shown noise exposure to have adverse effects on fetal hearing during late pregnancy [35,36]. There is, however, a possibility of that noise can have an adverse effect on hearing even during the embryonic period in the first trimester, when the auditory system is developing. The otocyst embryonic stem cells can produce hair cell-like cells [37], and these cells may react to loud sounds. According to these studies, it is not possible to indicate the stage of pregnancy when noise could harm the fetus [23]. We used the highest estimate in any pregnancy trimester as the exposure estimate.

### Strengths of the study

The main strengths of our study were the use of a validated measure of language acquisition and careful, blinded assessment of exposure to noise by an experienced occupational hygiene engineer. Expert assessment, based on both questionnaires and measurements, has been considered the best approach among exposure estimation methods [38]. Our assessment relied on information regarding earlier industrial hygiene measurements in the same or similar workplaces, women’s detailed descriptions of their working tasks, the level and duration of workplace noise exposure, and the women’s experience of disturbing noise. Self-reported data on occupational noise exposure is suggested to be a valid source when assessing exposure [39]. The background information on noise exposure and specific work tasks during pregnancy was collected several months before the mother filled out the ITC questionnaires on language acquisition. We therefore consider it unlikely that recall bias affected the reported noise exposure during pregnancy.

The ITC is designed for screening purposes, but it has also been used in research on language acquisition [40–42]. It has shown to be able to detect developmental growth and produce relatively stable rankings of children over short periods of time [43,44]. Määttä [42] also indicated that the individual differences in the development of prelinguistic skills showed rather high stability throughout the prelinguistic period. This suggests that even one measurement may give relatively permanent data on a child’s linguistic skills. Moreover, parent-report measures of communication and language skills have shown to be reliable and valid [45,46].

We considered several known determinants of language acquisition in the statistical analyses. In line with the literature, the older age of the mother and the child’s male gender were associated with lower language acquisition scores in our study, which indicates its validity [20]. Speaking, reading and singing to one’s child, in turn, were associated with high language acquisition scores in our data, which is also in line with earlier results [47].

This study focused on branches of work in which women are exposed to noise and obtained the data on these women from trade union registers. The women’s detailed descriptions of their work during pregnancy tasks made it possible to classify them accurately by occupation for the analyses. Thus, the information on occupation was reliable. The response rate was fairly good at baseline and when the children were 12 months old.

### Limitations of the study

However, our study has some limitations. Firstly, the number of mothers exposed to occupational noise of ≥85 dB was small, precluding analyses of higher exposure levels. Secondly, we could not separately analyze exposure to low frequency noise due to a lack of frequency-specific exposure data. Thirdly, we were not able to measure individual noise exposure. Thus, some misclassification of exposure may have occurred despite the exposure being assessed by an experienced occupational hygiene engineer, who used several data sources. In addition, the frequency of women in the highest exposure group declined in the last weeks of pregnancy (after 29 pregnancy weeks). If the most critical period is the last trimester with regard to language development, this could have introduced some misclassification of exposure. However, noise exposure was undoubtedly higher in the moderate/high exposure category than in the no-exposure group. Fourthly, we cannot totally rule out the fact that selective participation may have caused bias in the results. Furthermore, there is a possibility that our assessment measures did not reveal minor difficulties in language acquisition. Finally, at the age of one, language acquisition among children may vary considerably. For these reasons, the results regarding the effect of noise exposure on language acquisition are only suggestive.

## Conclusions

This is the first study to investigate the potential association between maternal noise exposure and children’s language acquisition. In general, our findings suggest no association between occupational noise exposure during pregnancy and language acquisition among one-year-old children. The children of kindergarten teachers exposed to human noise had lower language acquisition scores than the children of the non-exposed teachers, but this result should be interpreted with caution. Our suggestive findings warrant future studies by level and type of exposure.

## Supporting information

S1 Checklist *PLOS ONE* clinical studies checklist. (DOCX)

S2 Checklist STROBE statement—checklist of items that should be included in reports of observational studies. (DOCX)

S1 File (PDF)
