# Supplementary material for: Maternal occupational noise exposure during pregnancy and children’s early language acquisition
Source: PLoS One. 2024 Apr 16;19(4):e0301144. doi: 10.1371/journal.pone.0301144 (PMC11020523; doi:10.1371/journal.pone.0301144)
Supplement: S2 Checklist — (DOCX) [file pone.0301144.s002.docx]

STROBE Statement—checklist of items that should be included in reports of observational studies

|  | Item No. | Recommendation | Page  No. | Relevant text from manuscript |
| --- | --- | --- | --- | --- |
| **Title and abstract** | 1 | (*a*) Indicate the study’s design with a commonly used term in the title or the abstract | 2 | A cohort study |
|  |  | (*b*) Provide in the abstract an informative and balanced summary of what was done and what was found | 2 | A cohort study was conducted among women working in the food industry, as kindergarten teachers, musicians, dental nurses, or pharmacists who had a child aged <1 year. The analyses covered 408 mother-child pairs. Language acquisition was measured using the Infant-Toddler Checklist. An occupational hygienist assessed noise exposure individually as no (N=180), low (70–78 dB; N=108) or moderate/high exposure (>79 dB; N=120). |
| Introduction | | | |  |
| Background/rationale | 2 | Explain the scientific background and rationale for the investigation being reported | 4 | We hypothesized that regular exposure to noise during pregnancy might affect the fetal auditory system in ways that could influence language development. Thus, the aim of our study was to investigate the potential impact of a mother's occupational noise exposure during pregnancy on the language acquisition of her child. We investigated whether prenatal exposures to industrial noise, high frequency noise in dental clinics, human noise in kindergartens, and musical sounds in musicians’ work affect children's language acquisition at the age of one. The impact of maternal noise exposure during pregnancy on a child’s language acquisition has not been studied previously. |
| Objectives | 3 | State specific objectives, including any prespecified hypotheses | 4 |  |
| Methods | | | |  |
| Study design | 4 | Present key elements of study design early in the paper | 5 |  |
| Setting | 5 | Describe the setting, locations, and relevant dates, including periods of recruitment, exposure, follow-up, and data collection | 5–8 |  |
| Participants | 6 | (*a*) *Cohort study*—Give the eligibility criteria, and the sources and methods of selection of participants. Describe methods of follow-up  *Case-control study*—Give the eligibility criteria, and the sources and methods of case ascertainment and control selection. Give the rationale for the choice of cases and controls  *Cross-sectional study*—Give the eligibility criteria, and the sources and methods of selection of participants | 5–8 |  |
|  |  | (*b*) *Cohort study*—For matched studies, give matching criteria and number of exposed and unexposed  *Case-control study*—For matched studies, give matching criteria and the number of controls per case |  |  |
| Variables | 7 | Clearly define all outcomes, exposures, predictors, potential confounders, and effect modifiers. Give diagnostic criteria, if applicable | 5–8. 8–10 |  |
| Data sources/ measurement | 8* | For each variable of interest, give sources of data and details of methods of assessment (measurement). Describe comparability of assessment methods if there is more than one group | 5–8, 8–10 |  |
| Bias | 9 | Describe any efforts to address potential sources of bias | 12–13 |  |
| Study size | 10 | Explain how the study size was arrived at | 5–8, Fig 1 |  |

Continued on next page

| Quantitative variables | 11 | Explain how quantitative variables were handled in the analyses. If applicable, describe which groupings were chosen and why 5-12 |  |  |
| --- | --- | --- | --- | --- |
| Statistical methods | 12 | (*a*) Describe all statistical methods, including those used to control for confounding 10–12 |  |  |
|  |  | (*b*) Describe any methods used to examine subgroups and interactions 10–12 |  |  |
|  |  | (*c*) Explain how missing data were addressed 10–12 |  |  |
|  |  | (*d*) *Cohort study*—If applicable, explain how loss to follow-up was addressed  *Case-control study*—If applicable, explain how matching of cases and controls was addressed 8–12  *Cross-sectional study*—If applicable, describe analytical methods taking account of sampling strategy |  |  |
|  |  | (*e*) Describe any sensitivity analyses |  |  |
| Results | | | | |
| Participants | 13* | (a) Report numbers of individuals at each stage of study—eg numbers potentially eligible, examined for eligibility, confirmed eligible, included in the study, completing follow-up, and analysed 5–6 |  |  |
|  |  | (b) Give reasons for non-participation at each stage 5–6 |  |  |
|  |  | (c) Consider use of a flow diagram 6, Fig 1 |  |  |
| Descriptive data | 14* | (a) Give characteristics of study participants (eg demographic, clinical, social) and information on exposures and potential confounders 13–14, Table 1 |  |  |
|  |  | (b) Indicate number of participants with missing data for each variable of interest |  |  |
|  |  | (c) *Cohort study*—Summarise follow-up time (eg, average and total amount) |  |  |
| Outcome data | 15* | *Cohort study*—Report numbers of outcome events or summary measures over time |  |  |
|  |  | *Case-control study—*Report numbers in each exposure category, or summary measures of exposure 5–10, 13–14, Table 1 |  |  |
|  |  | *Cross-sectional study—*Report numbers of outcome events or summary measures |  |  |
| Main results | 16 | (*a*) Give unadjusted estimates and, if applicable, confounder-adjusted estimates and their precision (eg, 95% confidence interval). Make clear which confounders were adjusted for and why they were included, 14–20, Tables 2–5 |  |  |
|  |  | (*b*) Report category boundaries when continuous variables were categorized |  |  |
|  |  | (*c*) If relevant, consider translating estimates of relative risk into absolute risk for a meaningful time period |  |  |

Continued on next page

| Other analyses | 17 | Report other analyses done—eg analyses of subgroups and interactions, and sensitivity analyses none |  |  |
| --- | --- | --- | --- | --- |
| Discussion | | | | |
| Key results | 18 | Summarise key results with reference to study objectives 21–22 |  |  |
| Limitations | 19 | Discuss limitations of the study, taking into account sources of potential bias or imprecision. Discuss both direction and magnitude of any potential bias 24 |  |  |
| Interpretation | 20 | Give a cautious overall interpretation of results considering objectives, limitations, multiplicity of analyses, results from similar studies, and other relevant evidence 23–25 |  |  |
| Generalisability | 21 | Discuss the generalisability (external validity) of the study results 25 |  |  |
| Other information | |  | | |
| Funding | 22 | Give the source of funding and the role of the funders for the present study and, if applicable, for the original study on which the present article is based 26 |  | The funders had no role another than financial for the present study. |

*Give information separately for cases and controls in case-control studies and, if applicable, for exposed and unexposed groups in cohort and cross-sectional studies.

**Note:** An Explanation and Elaboration article discusses each checklist item and gives methodological background and published examples of transparent reporting. The STROBE checklist is best used in conjunction with this article (freely available on the Web sites of PLoS Medicine at http://www.plosmedicine.org/, Annals of Internal Medicine at http://www.annals.org/, and Epidemiology at http://www.epidem.com/). Information on the STROBE Initiative is available at www.strobe-statement.org.
